# When Software Becomes Medicine: Ignoring It May Soon Be Malpractice

**DOI:** 10.7759/cureus.82793

**Published:** 2025-04-22

**Authors:** Shaheen E Lakhan

**Affiliations:** 1 Department of Neurology, Global Neuroscience Initiative Foundation, Miami, USA; 2 Department of Neurology, A.T. Still University School of Osteopathic Medicine in Arizona, Mesa, USA; 3 Department of Neurology, Morehouse School of Medicine, Atlanta, USA; 4 Department of Neurology, Western University of Health Sciences, Pomona, USA

**Keywords:** artificial intelligence, closed-loop care, digital health, digital therapeutics, personalized medicine, prescription digital therapeutics, remote patient monitoring, software as a medical device

## Abstract

The convergence of mobile software, artificial intelligence, and biomedical science is ushering in a new era of precision medicine, defined not only by personalization but by real-time adaptability. This shift is exemplified by the evolution of Prescription Digital Therapeutics (PDTs): smartphone-based applications that treat a disease or condition and are regulated in the US by the FDA.

Early PDTs (PDT 1.0) such as reSET for substance use disorder, reSET-O for opioid use disorder, and Somryst for chronic insomnia digitized cognitive behavioral therapy (CBT) into a smartphone app and laid the foundation for regulated digital interventions. Now in the era of PDT 2.0, we see greater personalization, multimodal approaches beyond CBT, robust clinical validation, and targeting drug-like endpoints with Rejoyn for major depressive disorder, CT-132 for episodic migraine prevention, Luminopia for amblyopia, and CT-155 in development for negative symptoms of schizophrenia. Despite promising results, widespread adoption remains limited by reimbursement, clinician and patient awareness, and integration into clinical workflows. On the horizon is smart medicine: a convergence of traditional pharmacotherapy with PDTs that work together to enhance overall effectiveness, safety and tolerability, and engagement, adherence, and personalization. These medicines optimize outcomes in ways conventional therapies cannot and, in true closed-loop principles, assess for disease and symptoms and modulate both the digital and drug interventions.

Drawing from my work experience on multiple PDTs, this paper highlights how PDTs are transforming care. The FDA’s Prescription Drug Use-Related Software (PDURS) guidance affirms the regulatory foundation for integrating PDTs and drugs. Meaningful therapeutic advancements now depend not only on the molecular design but also on the mechanisms, digital or otherwise, that support its success. I postulate that within the next five years, omitting digital interventions when indicated will not just be outdated, it may be malpractice.

## Editorial

Over the last decade, my work experience across Sage Therapeutics, Fern Health, Lin Health, The Learning Corp, and Click Therapeutics has spanned the full life cycle of digital therapeutic innovation -- from design and development to clinical validation, regulatory strategy, and commercialization. These roles have offered unique insight into how digital interventions are reshaping medicine from within.

From digital therapy to prescription digital therapeutics (PDT 1.0)

For much of modern healthcare history, digital tools were relegated to adjunctive roles -- used primarily for wellness, tracking, or education. That changed in 2017, when the FDA granted its first marketing authorization for a prescription digital therapeutic (PDT): reSET (Pear Therapeutics, Inc., Boston, MA, USA), developed for substance use disorder. This marked the formal recognition that a smartphone application, when validated by clinical trials and regulatory scrutiny, could constitute medicine. Soon after, reSET-O (Pear Therapeutics, Inc.) extended this model to opioid use disorder in 2018 and Somryst (Pear Therapeutics, Inc.) to chronic insomnia in 2020. These products defined the PDT 1.0 era: software-based interventions derived from manualized cognitive-behavioral therapy, delivered via smartphones, and subject to FDA oversight.

However, the impact of PDT 1.0 was mixed. While reSET-O demonstrated early promise, an independent evaluation by the Institute for Clinical and Economic Review (ICER) concluded in 2022 that the evidence supporting its long-term clinical effectiveness and cost-effectiveness was limited [1]. These early PDTs pioneered the regulatory and commercial pathways but fell short of fully realizing the potential of digital medicine.

The rise of PDT 2.0: intelligent, validated, and (potentially) integrated therapeutics

Building upon the foundation of PDT 1.0, the second generation ushered in a new standard of digital therapeutics: multi-modal interventions with drug-like precision, dynamic personalization, and robust clinical validation. Unlike their predecessors, PDT 2.0 products target specific clinical endpoints that mirror those used in pharmacotherapy:

Rejoyn (Otsuka Precision Health, Inc, Princeton, NJ, USA), an FDA-authorized PDT under 510(k) pathway in 2024, improves symptoms of major depressive disorder (MDD) using the gold-standard Montgomery-Åsberg Depression Rating Scale (MADRS) in patients with incomplete response to antidepressants [2]. This mirrors the endpoint used in drug approvals such as brexpiprazole (Rexulti), reinforcing MADRS as a clinically validated measure in adjunctive MDD treatment.

CT-132 (Click Therapeutics, Inc., New York, NY, USA), an FDA-authorized PDT under De Novo pathway in 2025, reduces monthly migraine days (MMDs) in adults with episodic migraine in two Phase 3 studies [3]. Modern migraine drug trials, such as those for rimegepant (Nurtec ODT), have also used MMDs as their primary endpoint, underscoring the clinical validity of MMDs as a gold-standard measure.

Luminopia (Luminopia, Inc., Cambridge, MA, USA), an FDA-authorized PDT under De Novo pathway in 2021, treats amblyopia (lazy eye) in children by improving best-corrected visual acuity (BCVA) through immersive digital visual stimulation [4]. This contrasts with the traditional standard of care: occlusion therapy, where the stronger eye is patched to force the weaker eye to work harder. While effective, patching often suffers from poor adherence, psychosocial stigma, and discomfort.

Lastly, CT-155 (Click Therapeutics, Inc.), a PDT in late-stage clinical development with FDA Breakthrough Device Designation, targets experiential negative symptoms of schizophrenia, measured by the Clinical Assessment Interview for Negative Symptoms - Motivation and Pleasure subscale (CAINS-MAP) [5]. These symptoms represent a profoundly disabling domain of the illness for which there are currently no FDA-approved or authorized treatments, highlighting an area where PDTs may offer a novel path to address traditionally “undruggable” targets.

What distinguishes PDTs 2.0 are not only their advanced therapeutic mechanisms but also their integration into clinical workflows and real-world settings and their related rigorous evidence that meets the expectations of drug-level validation. 

Whereas the clinical validation and regulatory recognition of PDT 2.0 are rapidly advancing, real-world adoption has been limited. Key integration barriers include inconsistent payer coverage and a general lack of awareness by clinicians. Integration into existing clinical workflows remains complex, particularly in systems not equipped to support digital interventions alongside traditional pharmacotherapy. Additionally, the fragmented digital health landscape and variability in product quality have contributed to skepticism among providers and patients alike. Overcoming these challenges will require coordinated efforts across policy, education, and infrastructure to ensure that validated PDTs reach the patients who stand to benefit most.

The advent of smart medicine

While PDT 2.0 brought digital therapeutics into alignment with drug-level standards, the next frontier is even more transformative: the emergence of smart medicine. Defined as the convergence of traditional pharmacotherapy with PDTs, smart medicine represents a new category of treatment where software is not merely adjunctive, but actively enhances drug performance in a closed-loop system.

Smart medicines are also engineered to monitor symptoms, assess adherence, detect side effects, and modulate both the digital and pharmacologic components in response. This allows for dynamic optimization of combined treatment across efficacy, effectiveness, safety, tolerability, engagement, adherence, and personalization.

Rather than relying on episodic office visits or retrospective assessments, smart medicines operate continuously, adapting to patient behavior and context in real time. A migraine patient using CT-132, for example, could receive neurobehavioral interventions that respond to stress, sleep, or autonomic disruption, while their medication schedule is adjusted accordingly. Similarly, a patient on CT-155 may engage with neuromodulatory modules based on daily motivational patterns and negative symptom severity, supplementing pharmacologic strategies that often fall short in schizophrenia.

Smart medicine shifts the paradigm from reactive treatment to proactive, precision-guided care. It embodies the promise of closed-loop medicine: sensing, interpreting, and responding within a unified therapeutic system. This shift is also being codified in regulatory frameworks. The FDA’s 2023 draft guidance on Prescription Drug Use-Related Software (PDURS) explicitly recognizes software plus drug as part of the therapeutic paradigm. PDURS outlines when such software may require review and how it interacts with labeling and promotional standards, providing developers and clinicians with a clearer path for integrating digital into drug regimens. It validates what innovators have long asserted: that when software is married to a drug and generates additional clinical benefit, it becomes part of the medicine itself.

The ethical imperative: a new standard of care

Medicine evolves, but so too does the standard of care. Today, it would be unthinkable to manage stroke without neuroimaging, diabetes without hemoglobin A1C (HbA1C), or chest pain without EKG. Similarly, prescribing opioids without assessing misuse risk is now regarded as negligent rather than discretionary.

The same logic will soon apply to digital interventions. When a smart medicine comprising both pharmacologic and digital components is FDA-authorized, clinically validated, reimbursable, and proven to improve outcomes, failing to offer it may not just be a missed opportunity; it may breach professional duty.

Clinicians have an ethical obligation to practice to the standard of available evidence. As software becomes intrinsic to the function and effectiveness of medicine, neglecting its use in appropriate cases can no longer be considered acceptable. Smart medicines are not optional add-ons; they are central to optimizing care in chronic, complex, and underserved conditions.

As infrastructure, reimbursement, and regulatory frameworks align, the expectations of what constitutes best practice are rising. In that future, failure to use smart medicine when indicated will not merely reflect inertia, it may reflect malpractice.

## References

[1] (2025). Digital Health Technologies for Opioid Use Disorder: Final Evidence Report. https://icer.org/wp-content/uploads/2020/08/ICER_DHTs_for_OUD_Final_Report_052722.pdf.

[2] Rejoyn HCP (2025). Rejoyn Clinician Brief Summary. Clinician Brief Summary.

[3] Snipes C, Kuka A, Petrova M (2025). Efficacy and safety of a first-in-class investigational prescription digital therapeutic for episodic migraine (CT-132): phase 3 double-blind, randomized, controlled trial (P4-12.006). Neurology.

[4] (2025). De Novo Classification Request for Luminopia One. https://www.accessdata.fda.gov/cdrh_docs/reviews/DEN210005.pdf.

[5] (2025). A Study of CT-155 in Subjects With Schizophrenia (CONVOKE). NCT05838625. NCT05838625.

